# The Association Between Emotional Intelligence and Borderline Personality Disorder: A Meta‐Analysis

**DOI:** 10.1002/ijop.70092

**Published:** 2025-08-26

**Authors:** Angela Norris‐Nicholas, John M. Malouff, Jai Meynadier

**Affiliations:** ^1^ University of New England Armidale Australia

**Keywords:** association, borderline personality disorder, emotional intelligence meta‐analysis

## Abstract

This meta‐analysis synthesised findings on the association between emotional intelligence (EI) and borderline personality disorder (BPD) and evaluated potential moderators. Studies were sourced through systematic searches of four databases in April 2024. Studies had to report effect size data and participant numbers to be included in the meta‐analysis. The meta‐analysis included 25 samples and 7189 participants. A random effects model was used to calculate the overall effect size because this model allows inferences to studies not included in the analysis. Potential causes of heterogeneity were investigated through moderator analyses. Results showed that low EI was significantly associated with BPD (*r* = −0.41, *p* < 0.001, 95% CI [−0.51, −0.30]). Studies using diagnostic interviews had significantly higher effect sizes than those using self‐report scales. BPD was significantly associated with EI, whether EI was measured as an ability or as a trait, and whether BPD was assessed by diagnostic interview or self‐report. The main findings suggest that (1) individuals with BPD tend to have low EI, which typically involves difficulty perceiving, using, understanding and regulating emotions and (2) interventions that enhance EI are worth testing to evaluate whether they benefit individuals with BPD. This finding could help guide BPD interventions.

## 1 Introduction

Researchers have investigated whether low emotional intelligence may be related to Borderline Personality Disorder (BPD). *Emotional intelligence* is the ability to perceive, understand, use and manage emotions in oneself and others (Mayer et al. 2008). Evidence links low emotional intelligence to various types of psychopathology, psychological distress, interpersonal difficulties and substance abuse (Ahmadpanah et al. 2016; Brackett et al. 2004; Espinosa and Rudenstine 2020; Kun and Demetrovics 2010; Megías et al. 2018; Zhang et al. 2022).

Salovey and Mayer (1990) originally conceptualised emotional intelligence as an ability‐based concept akin to general intelligence. However, over the past 30 years, differing conceptualisations and definitions of emotional intelligence have arisen, leading to the development of various measures. As a result, two distinct models of emotional intelligence have emerged, differentiated by their measurement methods: the ability model and the trait model (O'Connor et al. 2019; Petrides 2011). The ability model, developed by Mayer et al. (2000, 2008), considers emotional intelligence to be a set of cognitive abilities involving perceiving, understanding, using and managing emotions in oneself and others. Consistent with this conceptualisation, *ability emotional intelligence* is measured through maximal performance tests that assess how proficiently people perform emotion‐related tasks (Bru‐Luna et al. 2021; O'Connor et al. 2019). The most prominent measure of ability emotional intelligence is the Mayer–Salovey–Caruso Emotional Intelligence Test (MSCEIT; Mayer et al. 2002, 2003), a 141‐item performance‐based measure that assesses four dimensions of emotional intelligence: perception, use, understanding and management of emotions.

The trait model, also known as the mixed model, encompasses Petrides and Furnham's (2001) conceptualisation of emotional intelligence as a set of personality traits and self‐perceived abilities and broader conceptualisations combining personality traits and emotional competencies such as interpersonal and intrapersonal skills. *Trait emotional intelligence* is typically assessed through self‐report questionnaires, measuring an individual's perceptions of their emotional capabilities (Bru‐Luna et al. 2021; O'Connor et al. 2019; Petrides 2011). Some measures, such as Bar‐On's EQ‐i (Bar‐On 2006), assess emotional intelligence combined with several other constructs (Brackett and Mayer 2003).

BPD is a severe mental health condition characterised by pervasive instability in emotions, identity and interpersonal relationships and impulsive, often self‐destructive, behaviour (American Psychiatric Association [APA] 2013). Diagnostic criteria for BPD (APA 2013) also include concerted efforts to avoid abandonment, intense anger, persistent feelings of emptiness, recurring suicidal behaviour and paranoid ideation or dissociative symptoms during stress. BPD is prevalent in approximately 1%–6% of the general population worldwide (Ellison et al. 2018).

Emerging during adolescence or early adulthood, BPD is associated with numerous adverse outcomes across the lifespan, including academic difficulties, recurrent job losses, relationship problems, substance abuse, self‐harm and suicide (Bagge et al. 2004; Grilo et al. 2021; Temes et al. 2019; Videler et al. 2019; Winsper et al. 2015). BPD is also linked to an increased risk for chronic disease and physical disability, increased healthcare use and reduced life expectancy (Grant et al. 2008; Jacobi et al. 2021; Powers and Oltmanns 2013; Quirk et al. 2016; Sansone et al. 2011; Smith et al. 2024; Temes et al. 2019; Zanarini et al. 2004). In addition, BPD significantly impacts those caring for individuals with the disorder, with studies showing that caregivers of people with BPD experience social and economic burden, psychological distress and mental health problems (Bailey and Grenyer 2013; Ekdahl et al. 2011; Goodman et al. 2011). Given the prevalence and substantial personal and societal costs associated with BPD, researchers have sought to identify factors associated with the disorder to inform treatment interventions.

The emotional aspects of BPD suggest that low emotional intelligence might be involved. Linehan's (Linehan and Keher 1993) proposed a biosocial model of the development of BPD, in which one element is a biological orientation toward very high emotional reactions. That emotional dysregulation fits theoretically with low emotional intelligence.

Numerous studies have examined the association between emotional intelligence and BPD. While many studies have found a negative relationship between emotional intelligence and BPD (e.g., Petrides et al. 2007), other studies have found a positive relationship (e.g., Lind et al. 2019). Furthermore, the strength of the association varies significantly across studies, ranging from *r* = −0.95 (Hurtado et al. 2016) to *r* = 0.09 (Beblo et al. 2010). These inconsistent findings could be due to methodological differences or sample characteristics. Researchers have adopted different approaches to conceptualising and measuring emotional intelligence across studies. Some researchers have measured emotional intelligence as a trait using self‐report measures (e.g., Gaher et al. 2013), while others have assessed emotional intelligence as an ability using performance‐based measures (e.g., Drummen 2016). Across studies, researchers have also varied in their approach to measuring BPD, with some researchers using diagnostic interviews to measure BPD (e.g., Peter et al. 2018) and others using psychometric scales to measure BPD traits (e.g., Leible and Snell Jr. 2004). Further, studies have examined the associations between emotional intelligence and BPD in different samples varying in age and gender. Consequently, it is difficult to draw firm conclusions about the level of association between emotional intelligence and BPD.

### 1.1 Aims and Hypotheses

Previous studies have reported inconsistent findings regarding the strength and direction of the relationship between emotional intelligence and BPD. A meta‐analysis could help remove the inconsistency, provide an overall level of association and help support the emotional‐dysregulation part of Linehan's biosocial model of BPD. The existence of an association between emotional intelligence and BPD is also important because it could help guide interventions for BPD.

This meta‐analysis aimed to synthesise study findings on the association between emotional intelligence and BPD. In addition, this meta‐analysis aimed to investigate potential moderators of the relationship between emotional intelligence and BPD, including the operationalisation of emotional intelligence (i.e., ability or trait), the type of emotional intelligence measure, the type of BPD measure and participants' gender and age. In light of the concerns surrounding the appropriateness of the EQ‐i in measuring many characteristics that are mixed with emotional intelligence (Brackett and Mayer 2003), as defined by Mayer et al. (2008), findings from the EQ‐i will be excluded from analyses when examining the relationship between emotional intelligence and BPD. It is hypothesised that emotional intelligence will be negatively associated with BPD. There were no a priori hypotheses about the potential moderators.

## 2 Method

### 2.1 Protocol and Registration

This meta‐analysis was conducted according to the Preferred Reporting Items for Systematic Reviews and Meta‐Analyses (PRISMA; Page et al. 2021) guidelines. The protocol for this meta‐analysis was registered with the International Prospective Register of Systematic Reviews (PROSPERO), registration number [omitted to preserve the author's identity].

### 2.2 Eligibility Criteria

Studies were included in the analysis if they reported an *r* or provided data that could be converted to an *r* (e.g., means of two diagnostic groups) for the association between emotional intelligence as defined by Mayer et al. (2000) and BPD (diagnosed or measured by self‐report). Studies also had to report participant numbers. There were no restrictions on publication dates, publication status, study location, type of sample or language of study. However, non‐English articles needed to be able to be translated into English using Google Translate to be included in the meta‐analysis. Studies were excluded if they did not measure at least two of the four dimensions of emotional intelligence as defined by Mayer et al. (2000, 2008): ability to perceive, understand, use and manage emotions in oneself and others. All the studies used measures that had evidence of reliability and validity.

### 2.3 Search Strategy

In April 2024, we conducted electronic searches for eligible studies within the following databases: EBSCOhost, ProQuest, PubMed and Scopus. The search terms used were (‘emotional intelligence’ OR ‘emotional quotient’) AND (‘borderline personality’). Searches were conducted within the article title and abstract fields in each database. To identify additional relevant research, we searched the reference lists of included studies and conducted forward citation searches on included studies in Google Scholar. We also emailed the corresponding author of the included studies to ask for any unpublished results that met the inclusion criteria of the meta‐analysis. No relevant unpublished results were obtained from any authors.

Two authors imported the search results into Covidence systematic review software and screened the articles by title and abstract and then full text. The second and third authors compared lists of studies and resolved any disagreement by consensus. The search was completed on June 1, 2024.

### 2.4 Data Extraction and Coding

We manually extracted data and coded each study for (1) author(s) and publication year, (2) country where the study was conducted, (3) sample size, (4) mean participant age, (5) female participant percentage, (6) sample type (nonclinical, clinical), (7) type of emotional intelligence (trait, ability), (8) emotional intelligence measure, (9) BPD measure, (10) type of BPD measure (diagnosis, scale), (11) effect size and (12) evidence of reliability and validity for all measures used. Data extracted to calculate the effect size of the association between emotional intelligence and BPD included correlations and independent group means with standard deviations. We used yes and no to code evidence of reliability and validity for the emotional intelligence and BPD measures used in each study.

Non‐English articles were translated into English using Google Translate. If a study had missing data or insufficient statistical information to compute an effect size, we emailed the corresponding author for the information needed. An effect size was obtained through this approach for one study (Petrides et al. 2007). Once the first researcher finished coding all studies, a second researcher checked the coding, with disagreements resolved by consensus. A third researcher then independently coded one‐third of the studies. Again, any disagreements between coders were resolved by consensus. Interrater agreement (reliability) between the two independent coders on codings for all effect size entries and moderators was 99% (κ = 0.99), indicating good data extraction and coding reliability.

### 2.5 Data Analysis

Analyses were performed using the Comprehensive Meta‐Analysis Version 4 software (CMA; Borenstein et al. 2022). CMA calculated a weighted Pearson's correlation coefficient, *r*, for the overall effect size between emotional intelligence and BPD. For nine studies that reported means and standard deviations instead of *r*, CMA converted these statistics to Pearson correlation coefficients for analysis. For two studies that administered two measures of emotional intelligence, CMA calculated the effect size by averaging the outcomes across both measures of emotional intelligence. We used a random‐effects model as we expected effect sizes to vary across studies due to differences in samples and measures. This model accounts for between‐study variance and allows for the results of the analysis to be generalised to comparable studies (Borenstein 2019; Borenstein et al. 2010).

## 3 Results

### 3.1 Results of Literature Search

#### 3.1.1 Study Selection

The literature search yielded 184 studies. Following the removal of 126 duplicates, 58 studies remained for screening. Although 30 studies appeared to meet the inclusion criteria, one study (Nagy Abdelhamid et al. 2021) was excluded because all participants had tramadol dependence, potentially confounding the results. Four other studies (Avarzamani et al. 2021; Bakhshizadeh et al. 2019; Pirkhaefi et al. 2015; Webb and McMurran 2008) were excluded because they only used the EQ‐i scale, which measures a broad array of constructs while not explicitly measuring at least two facets of emotional intelligence as defined by Mayer et al. (2008). An additional study (Janke et al. 2018) was excluded because the only emotional intelligence measure used was the Test of Emotional Intelligence (TEMINT; Schmidt‐Atzert and Bühner 2002), which assesses only one facet of emotional intelligence as defined by Mayer and Salovey (2008).

**FIGURE 1:**
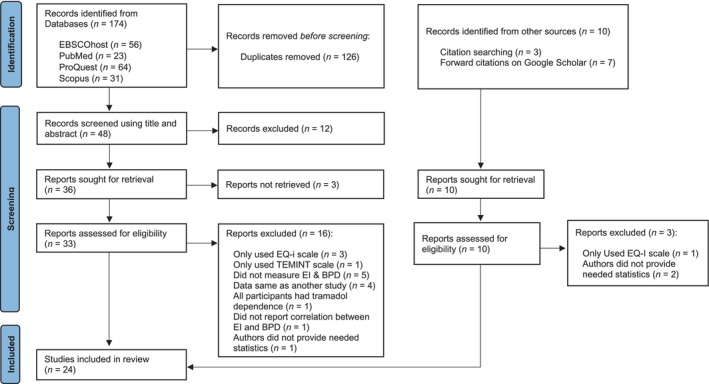
PRISMA flow diagram of study selection. EI, emotional intelligence; EQ‐i, Emotional Quotient Inventory; *n*, number; TEMINT, Test of Emotional Intelligence.

Information about the study selection process is presented in a PRISMA flow diagram (Moher et al. 2009) in Figure 1. The data file used to run the analysis is located at [location concealed to maintain author anonymity].

#### 3.1.2 Study Characteristics

Table 1 shows key study characteristics and effect sizes. The 25 independent samples analysed in the meta‐analysis included 7189 participants (mean female percentage 68%, mean age = 26 years). All studies were cross‐sectional.

**TABLE 1:** Information about samples included in the meta‐analysis.

Study	*N*	Sample	% female	Mean age	Country	Type of EI	EI measure	Type of BPD measure	BPD measure	*r*
Beblo et al. (2010)^g^	39	Clinical—patients and HC	85	30	Germany	Ability	MSCEIT	Diagnosis	SCID‐II DSM‐4	0.09
Christodoulou (2016)—High School^g^	359	Nonclinical—adolescents	45	15	Cyprus	Trait	TEIQue	Scale	BPFSC	0.01
Christodoulou (2016)—Junior High^g^	196	Nonclinical—adolescents	46	13	Cyprus	Trait	TEIQue	Scale	BPFSC	−0.06
Drummen (2013)^d^, ^g^	55	Clinical—patients and HC	69	35	Netherlands	Ability	MSCEIT	Diagnosis	SCID‐II DSM‐4	−0.59
Gaher et al. (2013)	579	Nonclinical—university students	70	20	US	Trait	SEIS	Scale	PAI‐BOR	−0.45
Gardner and Qualter (2009)	523	Nonclinical—adults	78	34	UK	Ability, Trait	MSCEIT, SEIS	Scale	PAI‐BOR, MSI‐BPD, PDQ‐4 BOR	−0.46
Hertel et al. (2009)^c^, ^f^	113	Clinical—patients and HC	73	36	Germany	Ability	MSCEIT	Diagnosis	SCID‐II DSM‐4	−0.86
*Holaway (2008)^b^, ^g^	120	Nonclinical—university students	59	19	US	Ability, Trait	MSCEIT, TMMS	Scale	MSI‐BPD	−0.24
Hurtado et al. (2016)^g^	33	Clinical—patients and HC	—	—	Spain	Ability	MSCEIT	Diagnosis	SCID‐V DSM‐5	−0.95
Janczak (2021)^g^	431	Nonclinical—adults	58	30	Poland	Trait	TEIQue	Scale	BPI	−0.37
Khosravi and Hassani (2022)^g^	110	Clinical—patients	38	30	Iran	Trait	TMMS	Scale	BPDSI	−0.56
Krajinak et al. (2018)	242	Nonclinical—university students	74	19	US	Trait	TEIQue	Scale	SNAP‐2	−0.55
Leible and Snell Jr. (2004)^b^	1359	Nonclinical—university students	57	—	US	Trait	TMMS	Scale	PDQ‐4+ BOR	−0.22
Lind et al. (2019)^g^	60	Clinical—patients and HC	93	29	Denmark	Ability	MSCEIT	Diagnosis	SCID‐II DSM‐4	0.04
Lizeretti et al. (2012)^c^, ^g^	177	Clinical—patients and HC	76	27	Spain	Trait	TMMS‐24	Diagnosis	SCID‐I DSM‐4	−0.11
Martskvishvili and Mestvirishvili (2014)^g^	120	Nonclinical—university students	72	19	Georgia	Trait	TEIQue	Scale	PDQ‐4+ BOR	−0.29
Mashhadi et al. (2010)^g^	358	Nonclinical—university students	68	22	Iran	Trait	TMMS	Scale	STQ‐STB	−0.36
Peter et al. (2013)^d^, ^g^	309	Clinical—patients and HC	67	34	Netherlands	Ability	MSCEIT	Diagnosis	SCID‐II DSM‐4	0.07
Peter et al. (2018)^e^, ^g^	132	Clinical—patients and HC	73	35	Netherlands	Ability	MSCEIT	Diagnosis	SCID‐II DSM‐4	−0.50
Petrides et al. (2007)—study 3^a^, ^g^	212	Nonclinical—university students	83	23	Spain	Ability	TEIQue	Scale	IPDE	−0.57
Ruiz et al. (2012)^g^	354	Nonclinical—mostly university students	71	26	Spain	Trait	TMMS‐24	Scale	CEPER‐III‐BPD	−0.07
Sheinin (2018)	117	Nonclinical—university students	85	22	UK	Ability	MSCEIT	Scale	PDQ‐4+ BOR	−0.12
Sinclair and Feigenbaum (2012)	72	Clinical—patients and HC	85	—	UK	Trait	TEIQue	Diagnosis	Existing diagnosis	−0.87
Sneesby (2023)	504	Nonclinical—university students	64	19	US	Trait	SEIS	Scale	PAI‐BOR	−0.28
Torenli Kaya et al. (2023)^g^	615	Nonclinical—adults	50	35	Turkey	Trait	TEIQue‐SF	Scale	BPI	−0.47

Abbreviations: BPDSI, Borderline Personality Disorder Severity Index; BPFSC, Borderline Personality Features Scale; BPI, borderline personality inventory; CEPER‐III‐BPD, Exploratory Questionnaire of Personality‐III—Borderline Personality Scale; EI, emotional intelligence; HC, healthy controls; IPDE, International Personality Disorder Examination; MSCEIT, Mayer Salovey‐Caruso Emotional Intelligence Test; MSI‐BPD, MacLean Screening Instrument for *BPD; N, s*ample size; PAI‐BOR, Personality Assessment Inventory‐Borderline Features Scale; PDQ‐4 BOR, Personality Diagnostic Questionnaire Version 4—Borderline Personality Scale; PDQ‐4+ BOR, Personality Diagnostic Questionnaire Version 4+ − Borderline Personality Scale; *r*, Pearson *r* correlation coefficient; SCID‐I DSM‐4, Structured Clinical Interview for DSM‐IV Axis 1 Disorders; SCID‐II DSM‐4, Structured Clinical Interview for DSM‐IV Axis 2 Personality Disorders; SCID‐5 DSM‐5, Structured Clinical Interview for DSM‐V; SEIS, Schutte Emotional Intelligence Scale; SNAP‐2, The Schedule for Nonadaptive and Adaptive Personality‐2; STQ‐STB, Psychotic Traits Questionnaire—Borderline Personality Scale; TEIQue, Trait Emotional Intelligence Questionnaire; TEIQue‐SF, Trait Emotional Intelligence Questionnaire Short Version; TMMS, Trait Meta‐Mood Scale; TMMS‐24, Trait Meta‐Mood Scale Short Version.

^a^ Correlation not reported in the article but obtained through emailing the corresponding author.

^b^ Total emotional intelligence score not reported in article but was obtained through averaging subscale scores.

^c^ Controlled for gender and age.

^d^ Controlled for gender, age and education.

^e^ Controlled for gender, age, education and IQ.

^f^ BPD group comprised only females, but control group comprised males and females.

^g^ Study conducted in language other than English.

### 3.2 Quality Assessment

The quality of research in the meta‐analysis was assessed by evaluating whether studies used reliable and valid measures of emotional intelligence and BPD. The assessment showed that all studies included in the meta‐analysis used measures with published evidence of reliability and validity.

### 3.3 Overall Effect Size

**FIGURE 2:**
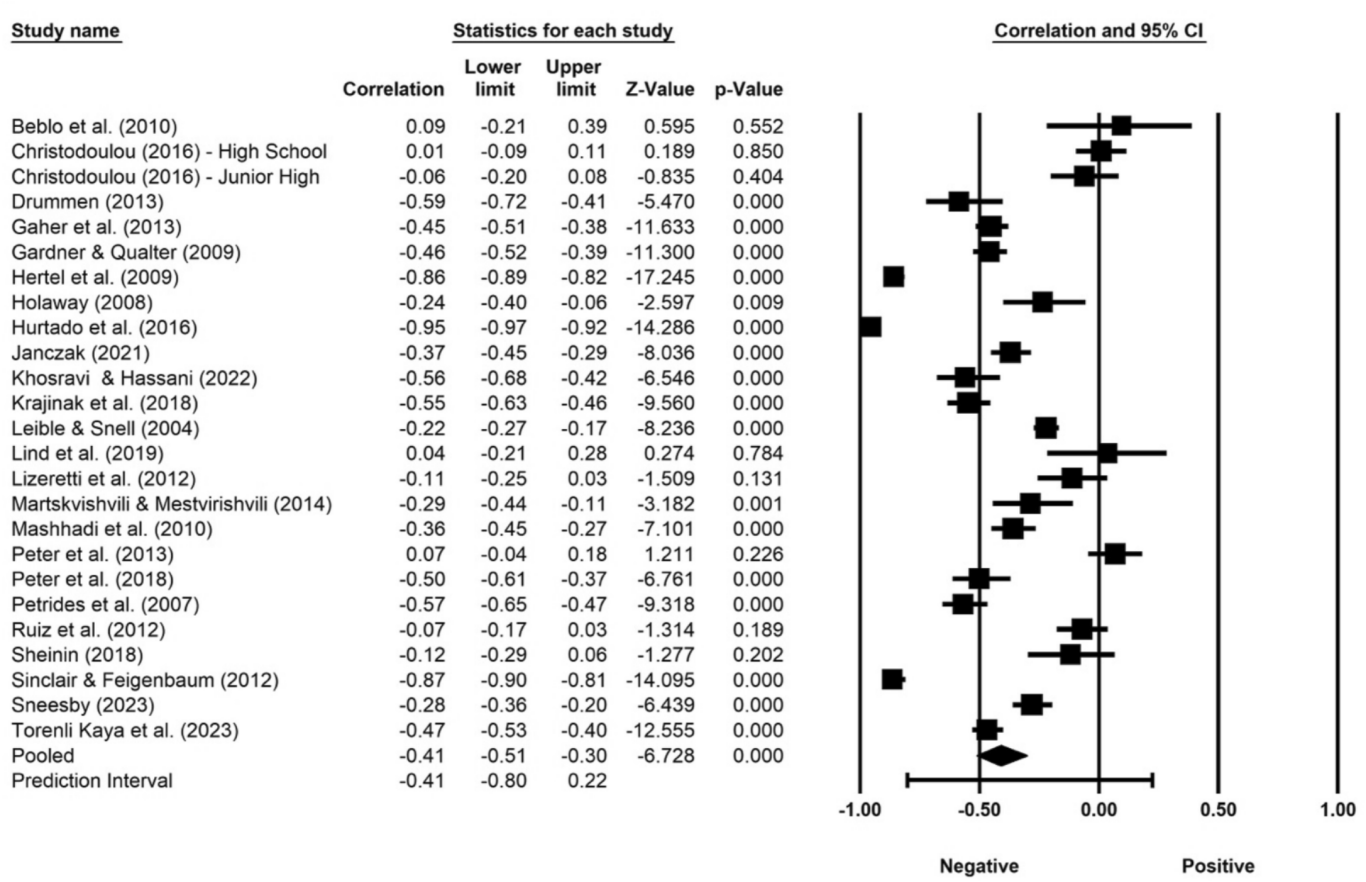
Forest plot of the meta‐analysis of correlations between emotional intelligence and BPD.

The random effects model used to conduct the meta‐analysis showed a significant negative correlation (effect size) between emotional intelligence and BPD (*r* = −0.41, 95% CI [−0.51, −0.30], *p <* 0.001, *k* = 25). Figure 2 shows the forest plot of effect sizes for all samples included in the meta‐analysis. The size of the box is proportionate to the weight of the study in relation to the overall weighted association.

### 3.4 Heterogeneity Analyses

There was high heterogeneity in effect sizes across the samples; *Q*(24) = 693.60, *p* < 0.001, *T* ^2^ = 0.099, *I* ^2^ = 0.97. The high *I* ^2^ index indicated that 97% of the observed variance reflected variance in true effects rather than chance, justifying moderator analyses to examine possible moderators of effect size (Bloch 2014).

### 3.5 Sensitivity Analyses

Sensitivity analyses using the one study removed method (repeating analyses while eliminating one study at a time) demonstrated that none of the included studies significantly impacted the meta‐analytic effect size. When any individual study was excluded from the analysis, the meta‐analytic effect size remained significant (*p* < 0.001) and within the 95% confidence interval of the meta‐analytic effect size calculated from the inclusion of all studies, indicating the robustness of the results.

### 3.6 Publication Bias

**FIGURE 3:**
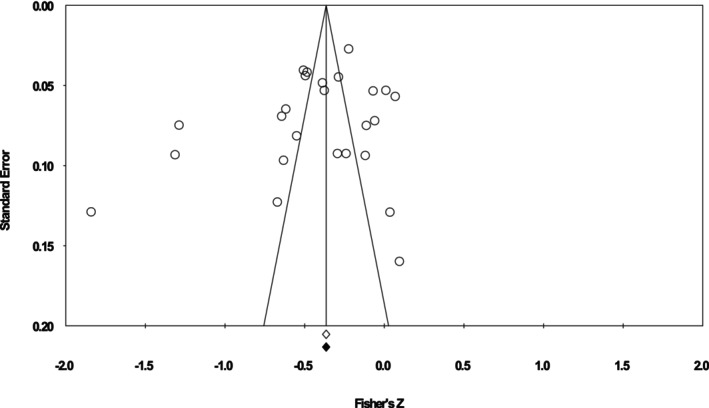
Funnel plot of standard error by Fisher's Z.

Figure 3 shows the funnel plot of effect sizes for all samples included in the meta‐analysis. Visual inspection of the funnel plot shows a symmetrical distribution, indicating no evidence of publication bias. Egger's test for asymmetry of the funnel plot was not significant (*p* = 0.20, two‐tailed); Begg's rank correlation test was not significant (*p* = 0.61, two‐tailed), suggesting no evidence of publication bias. Further, Duval and Tweedie's trim and fill method found no evidence of missing studies and did not recommend imputing any studies. Together, these results suggest that it is unlikely that the meta‐analytic effect size was impacted by publication bias.

### 3.7 Moderator Analyses

#### 3.7.1 Subgroup Analyses

Subgroup analyses investigated whether the meta‐analytic effect size was moderated by categorical variables such as the type of emotional intelligence (ability or trait), the emotional intelligence measure, whether the sample was clinical and the BPD measure. Table 2 presents the results of the subgroup analyses of categorical variables. Sixteen different BPD measures were used across the 25 samples, resulting in less than two samples in most categories, preventing moderator analyses of individual BPD measures.

**TABLE 2:** Subgroup analyses of categorical variables.

			(95% CI)			
Moderator	*k*	*r*	Lower	Upper	*p*	*T* ^2^
Type of emotional intelligence, *Q*_B_(1) = 0.84, *p* = 0.36						
Ability	8	−0.49	−0.65	−0.29	< 0.001	0.110
Trait	15	−0.38	−0.51	−0.22	< 0.001	0.110
Clinical sample, *Q*_*B*_(1) = 2.01, *p* = 0.16						
Yes	10	−0.55	−0.40	−0.22	< 0.001	0.410
No	15	−0.31	−0.77	−0.22	0.002	0.033
Emotional intelligence measure, *Q*_B_(3) = 1.58, *p* = 0.66						
MSCEIT	8	−0.49	−0.66	−0.27	< 0.001	0.130
TEIQue	8	−0.44	−0.62	−0.22	< 0.001	0.130
SEIS	2	−0.37	−0.71	0.12	0.132	0.130
TMMS	5	−0.27	−0.54	0.04	0.087	0.130
Type of BPD measure, *Q*_B_(1) = 4.35, *p* = 0.04						
Diagnostic interview	9	−0.55	−0.68	−0.39	< 0.001	0.097
Self‐report scale	16	−0.33	−0.46	−0.18	< 0.001	0.097

The Q values in Table 2 show the significance of the group difference. Subgroup analyses revealed that the effect size did not significantly differ depending on whether emotional intelligence was operationalised as an ability or a trait. The effect size also did not significantly differ depending on the type of emotional intelligence measure used, with the MSCEIT and TEIQue scales showing significant moderate negative correlations and the SEIS and TMMS showing nonsignificant weak negative correlations. These results indicate that neither the type of emotional intelligence assessed (ability or trait) nor the emotional intelligence measure used moderated the association between emotional intelligence and BPD. The type of sample (clinical or not) was not a significant moderator. The potential moderators of clinical sample or not and type of measure (interview or self‐report) overlapped extensively, with almost all clinical groups receiving diagnosis based on an interview.

Subgroup analyses examining the moderating effect of the type of BPD measure found that the effect size significantly differed depending on whether BPD was measured by diagnosis or scale, with samples that used diagnostic interviews showing a significant moderate to strong negative correlation and samples that used psychometric scales showing a significant but weaker negative correlation.

#### 3.7.2 Meta‐Regression

Meta‐regression revealed that neither the percentage of females in the sample (coefficient = −0.003, 95% CI [−0.012, 0.005], *p* = 0.46) nor the mean age of the sample (coefficient = −0.016, 95% CI [−0.033, 0.001], *p* = 0.06) was a significant moderator of effect size.

## 4 Discussion

This meta‐analysis is the first to synthesise findings on the association between emotional intelligence and BPD. Findings from 25 samples and 7189 participants showed a significant negative association between emotional intelligence and BPD (*r* = −0.41), supporting the hypothesis that lower emotional intelligence would be associated with BPD. These findings suggest that individuals with BPD have deficits in perceiving, understanding, using and managing emotions. Sensitivity analyses showed that no individual study significantly influenced the overall effect size, and publication bias analyses indicated the results were unlikely to be impacted by publication bias, suggesting the findings are robust. According to Cohen's (1988) criteria, the observed effect size of −0.41 is medium. The association found in the present meta‐analysis was similar to the −0.36 association between emotional awareness (which is a component of emotional intelligence) and BPD reported in a meta‐analysis by Derks et al. (2017). The present findings align with previous research findings showing that individuals with BPD have deficits in specific facets of emotional intelligence, such as perceiving, understanding, using and regulating emotions (Beblo et al. 2013; Daros and Williams 2019; Mitchell et al. 2014; Salgado et al. 2020). Further, the present results are consistent with Linehan's (1993) biosocial theory, which posits that emotional dysregulation is a core characteristic of BPD.

High heterogeneity in effect sizes across studies indicated potential moderators of effect size. No moderator effects were found for either the type of emotional intelligence assessed or the emotional intelligence measure used, suggesting that the relationship of emotional intelligence and BPD is robust across different types and measures of emotional intelligence.

Diagnostic interviews yielded a significantly higher effect size than self‐report scales. These findings are consistent with previous studies and meta‐analyses showing that diagnostic interviews and self‐report scales often yield different results when assessing BPD (e.g., Hasler et al. 2014; Hopwood et al. 2008). The effect‐size differences between diagnostic interviews and self‐report scales may be due to differences between clinical and nonclinical samples. In the studies included in the present meta‐analysis, diagnostic interviews were conducted in clinical samples, which consisted of BPD patients who had been referred for treatment or were already receiving treatment for BPD. Consequently, clinical samples may capture more severe presentations of the disorder, where deficits in emotional intelligence are more pronounced. In contrast, self‐report scales were used to screen for borderline personality traits in the general population and, therefore, may capture milder subclinical presentations of the disorder (Gardner and Qualter 2009). Hence, the present findings could indicate that the association between emotional intelligence and BPD is stronger among individuals with more severe symptoms.

However, the meta‐analytic differences between diagnostic interviews and self‐report scales may also be due to methodological differences between the two measures. The diagnostic interviews were structured or semi‐structured clinical interviews administered by trained clinicians. These interviews are considered the ‘gold standard’ for diagnosing BPD and involve a comprehensive assessment of symptom severity and impairment against strict diagnostic criteria (Biskin and Paris 2012; Hasler et al. 2014). In contrast, the self‐report scales were questionnaires that relied on participants' self‐perceptions of their BPD symptomology. Self‐report measures are vulnerable to response biases, such as social desirability bias, where individuals underreport less socially desirable attributes and overreport more socially desirable ones to present themselves more favourably (Hasler et al. 2014; Wetzel et al. 2013). Research shows that self‐report measures can be particularly unreliable when assessing individuals with BPD, as they may have poor self‐awareness and retrospective memory and often underreport the frequency and severity of their symptoms on self‐report questionnaires (Hasler et al. 2014; Mneimne et al. 2021).

Ability measures of emotional intelligence had a significant meta‐analytic association with BPD. That association was somewhat higher than for the association between trait emotional intelligence and BPD, but the difference in level of association was not significant. The similarity of the associations suggests that lower emotional intelligence, whether measured as ability or trait, is associated with BPD.

Several limitations are relevant when interpreting the results. First, only cross‐sectional studies were included in this meta‐analysis, preventing causal inferences about the relationship between low emotional intelligence and BPD. It may be that low emotional intelligence leads to the development and maintenance of BPD or that BPD leads to lower emotional intelligence, or that some third variable leads to both. Second, several potential moderators were not investigated in this meta‐analysis. Factors such as comorbid mental health conditions, adverse life events, psychiatric medication, race‐ethnicity, education and general intelligence were reported as potential moderators in some of the included studies. However, because only some studies reported this information, we could not explore the moderating effects of these variables on the association between emotional intelligence and BPD. Third, the nonsignificant findings in the present moderator analyses could be due to low statistical power rather than the absence of a moderator effect.

The finding that individuals with BPD have low emotional intelligence might have implications for treatment approaches. Meta‐analytic evidence suggests that emotional intelligence can be improved through training (Hodzic et al. 2018; Hubscher‐Davidson et al. 2021; Mattingly and Kraiger 2019; Schutte et al. 2013).

Some existing treatments for BPD, like dialectical behaviour therapy (DBT; Linehan 1993), already incorporate at least one element of emotional intelligence: how to manage one's own emotions. Mentalisation‐based treatment for BPD involves training clients to visualise the emotions of others (Batemen and Fonagy 2010). Emotional‐intelligence training could add other elements that may be valuable for treatment of BPD, such as how to help others regulate their emotions. Individuals with BPD can show behaviour that leads others to have strong negative reactions and to shy away (Ahmed et al. 2021). The ability to help others feel less negative could be valuable to individuals with BPD. Further, training in a broad range of emotional‐intelligence skills might have more impact than training in one or two. However, the value of emotional‐intelligence training in BPD and its advantages over current BPD treatments have yet to be adequately evaluated. Jahangard et al. (2012) found that BPD patients who received emotional‐intelligence skills training showed significant improvements in emotional intelligence. It remains unclear whether training in emotional‐intelligence skills improves functional outcomes of individuals with BPD.

The present findings provide a basis for randomised controlled trials (RCTs) to investigate further the benefits of emotional intelligence training as a treatment for BPD. Future studies could use both diagnostic interviews and self‐report scales to clarify the moderating effects of these measures. Future research could also examine the potential moderators not investigated in this meta‐analysis to provide a better understanding of the relationship between emotional intelligence and BPD.

## 5 Conclusion

In summary, the findings from this meta‐analysis suggest that the association between emotional intelligence and BPD is robust across different types and measures of emotional intelligence, age groups and genders. The consistency of these results across different researchers, countries, time points and samples further underscores the robustness of the findings.

## Author Contributions

Norris‐Nicholas co‐completed study search, she entered data, she analysed the data, and she wrote a draft of the article. Malouff had the idea for the meta‐analysis, checked every data entry, checked results of analyses, and edited the manuscript. Meynadier co‐completed the study search, independently entered 1/3 of key data, and made comments about the manuscript.

## Ethics Statement

The authors complied with ethical standards. There was no contact by these researchers with participants in studies included in the meta‐analysis.

## Consent

The authors have nothing to report.

## Conflicts of Interest

The authors declare no conflicts of interest.

## Data Availability

The data that support the findings of this study are openly available in OSF at https://osf.io/dashboard, reference number https://osf.io/25mqd/.
